# PCA-PAM50 improves consistency between breast cancer intrinsic and clinical subtyping reclassifying a subset of luminal A tumors as luminal B

**DOI:** 10.1038/s41598-019-44339-4

**Published:** 2019-05-28

**Authors:** Praveen-Kumar Raj-Kumar, Jianfang Liu, Jeffrey A. Hooke, Albert J. Kovatich, Leonid Kvecher, Craig D. Shriver, Hai Hu

**Affiliations:** 1 Chan Soon-Shiong Institute of Molecular Medicine at Windber, Windber, PA USA; 2 Clinical Breast Care Project, Murtha Cancer Center Research Program, Uniformed Services University of Health Sciences/Walter Reed National Military Medical Center, Bethesda, MD USA; 3 Murtha Cancer Center Research Program, Uniformed Services University of Health Sciences/Walter Reed National Military Medical Center, Bethesda, MD USA

**Keywords:** Breast cancer, Breast cancer, Data processing, Data processing

## Abstract

The PAM50 classifier is widely used for breast tumor intrinsic subtyping based on gene expression. Clinical subtyping, however, is based on immunohistochemistry assays of 3–4 biomarkers. Subtype calls by these two methods do not completely match even on comparable subtypes. Nevertheless, the estrogen receptor (ER)-balanced subset for gene-centering in PAM50 subtyping, is selected based on clinical ER status. Here we present a new method called Principle Component Analysis-based iterative PAM50 subtyping (PCA-PAM50) to perform intrinsic subtyping in ER status unbalanced cohorts. This method leverages PCA and iterative PAM50 calls to derive the gene expression-based ER status and a subsequent ER-balanced subset for gene centering. Applying PCA-PAM50 to three different breast cancer study cohorts, we observed improved consistency (by 6–9.3%) between intrinsic and clinical subtyping for all three cohorts. Particularly, a more aggressive subset of luminal A (LA) tumors as evidenced by higher *MKI67* gene expression and worse patient survival outcomes, were reclassified as luminal B (LB) increasing the LB subtype consistency with IHC by 25–49%. In conclusion, we show that PCA-PAM50 enhances the consistency of breast cancer intrinsic and clinical subtyping by reclassifying an aggressive subset of LA tumors into LB. PCA-PAM50 code is available at ftp://ftp.wriwindber.org/.

## Introduction

Breast cancer (BC) is one of the few tumor types with established molecular classification and targeted treatment regimen that yield improved clinical outcomes^1–4^. There are four widely-accepted intrinsic subtypes based on PAM (Prediction Analysis of Microarray) 50^5^ gene expression profiles: Luminal A (LA), Luminal B (LB), Her2-enriched (Her2) and basal-like (Basal). The originally defined normal-like^1^ (Normal) breast cancer subtype is now less frequently used^6–8^. Clinical subtyping of BC is based on immunohistochemistry (IHC) assays for the estrogen receptor (ER), progesterone receptor (PR), and human epidermal growth factor receptor 2 (HER2). More institutions now include Ki67, thus classifying tumors into triple-negative (TN; ER−/PR−/HER2−), HER2+ (ER−/PR−/HER2+), LA (ER+/HER2−/Ki67−), LB1 (ER+/HER2−/Ki67+) and LB2 (ER+/HER2+)^9–12^. For clarity, we will refer to clinical subtyping as IHC subtyping. IHC subtyping is the only accepted molecular assay for patient treatment decision-making^13–17^. BC intrinsic and clinical subtypes do not completely match even when comparing intra-subtypes, especially for LB^18^. There is limited research in the literature attempting to increase the consistency of intrinsic and IHC subtypes^8,19^.

Accurate classification of tumors based on gene expression data is not a trivial task, and it lacks standard practices. The PAM50^5^ classifier, which is also deployed in *Genefu* Bioconductor package^20^, makes calls based on the 50 gene centroid correlation distance to LA, LB, Basal, Her2 and normal-like centroids. However, the application of the PAM50 algorithm has its challenges. The two main challenges are (1) balancing ER status and (2) the gene centering procedures^5,21^. The PAM50 classifier works accurately if the original cohort/dataset is ER status-balanced. However this is often not the case with most genome-wide studies. In such cases, a conventional strategy is to choose a subset which is ER status-balanced and use the median derived from that subset to gene center the entire cohort. In practice, an ER-balanced subset is chosen based on IHC-defined ER status. There have been reports that IHC-defined ER status, which is based on protein expression, not being completely consistent with ER status defined by gene expression^22,23^. This inconsistency may impact the accuracy of the subsequent gene centering procedure which is aimed to minimize the bias of the dynamic range of the expression profile per sequencing technology. As a result, such inconsistency may contribute to the discrepancy between the IHC and PAM50 subtyping results. Hence, we explored the possibility of using a gene expression-based ER-balanced subset for gene centering leveraging principal component analyses (PCA) and iterative PAM50 calls to avoid introducing protein expression-based data into a gene expression-based subtyping method. We validated our method termed PCA-PAM50 using three different primary breast tumor datasets: an in-house 118 patients cohort, The Cancer Genome Atlas (TCGA)^24^ Breast cancer RNA-Seq 1097 patients cohort obtained from the Genomic Data Commons [https://gdc.cancer.gov/], and the Molecular Taxonomy of Breast Cancer International Consortium (METABRIC)^25^ discovery set of 997 patients. Our method resulted in improved consistency between PAM50 calls and IHC subtypes compared to the conventional method for all three cohorts, and this improved consistency is attributable to re-classification of an aggressive subset of LA tumors as LB.

## Materials and Methods

### In-house RNA-Seq dataset

118 primary breast tissue samples from consented patients were obtained through the Clinical Breast Care Project (CBCP)^26^, using HIPAA-compliant, IRB-approved protocols. Tumor cells were collected by laser microdissection (LMD) and RNA extracted using the illustra triplePrep Kit (GE Healthcare). Paired-end mRNA sequencing was performed using the Illumina HiSeq platform by a vendor (NantOmics, LLC, Culver City, CA, USA) and preprocessed using PRINSEQ version 0.20.4^27^ to remove duplicate reads, trim low-quality bases (20 and less) and poly A/T/N tails; the minimum length retained was 35 nt. GSNAP version 2016–08–24^28,29^ was used for splice alignment to reference genome hg38 from ENSEMBL release 85^30^. HTSeq^31^ was used to quantify gene expression with the guidance of gene annotation file GTF from ENSEMBL release 85. Pipelines were developed in PERL and R languages. Gene expression was upper-quartile normalized.

IHC subtypes for in-house dataset were derived using the IHC assays for ER, PR, HER2, and Ki67 in a centralized CLIA-certified laboratory following standardized protocols. A tumor was considered ER or PR positive if the corresponding nuclear staining was >1% by the American Society of Clinical Oncology (ASCO) and College of American Pathologists (CAP) guidelines^32^. The HER2 result was considered negative if IHC = 0 or 1+ and positive if IHC = 3+. For IHC = 2+, Florescence *In-Situ* Hybridization (FISH) was used to determine the final HER2 status. Ki67 was positive if nuclear staining was ≥15%^11,16^. IHC subtypes were defined for triple-negative (TN; ER−/PR−/HER2−), HER2+ (ER−/PR−/HER2+), LA (ER+/HER2−/Ki67−), LB1 (ER+/HER2−/Ki67+) and LB2 (ER+/HER2+)^9–12^. There were 30 TN, 16 HER2+, 16 LA, 39 LB1, and 17 LB2 in this cohort.

### TCGA RNA-Seq data

**Figure 1:**
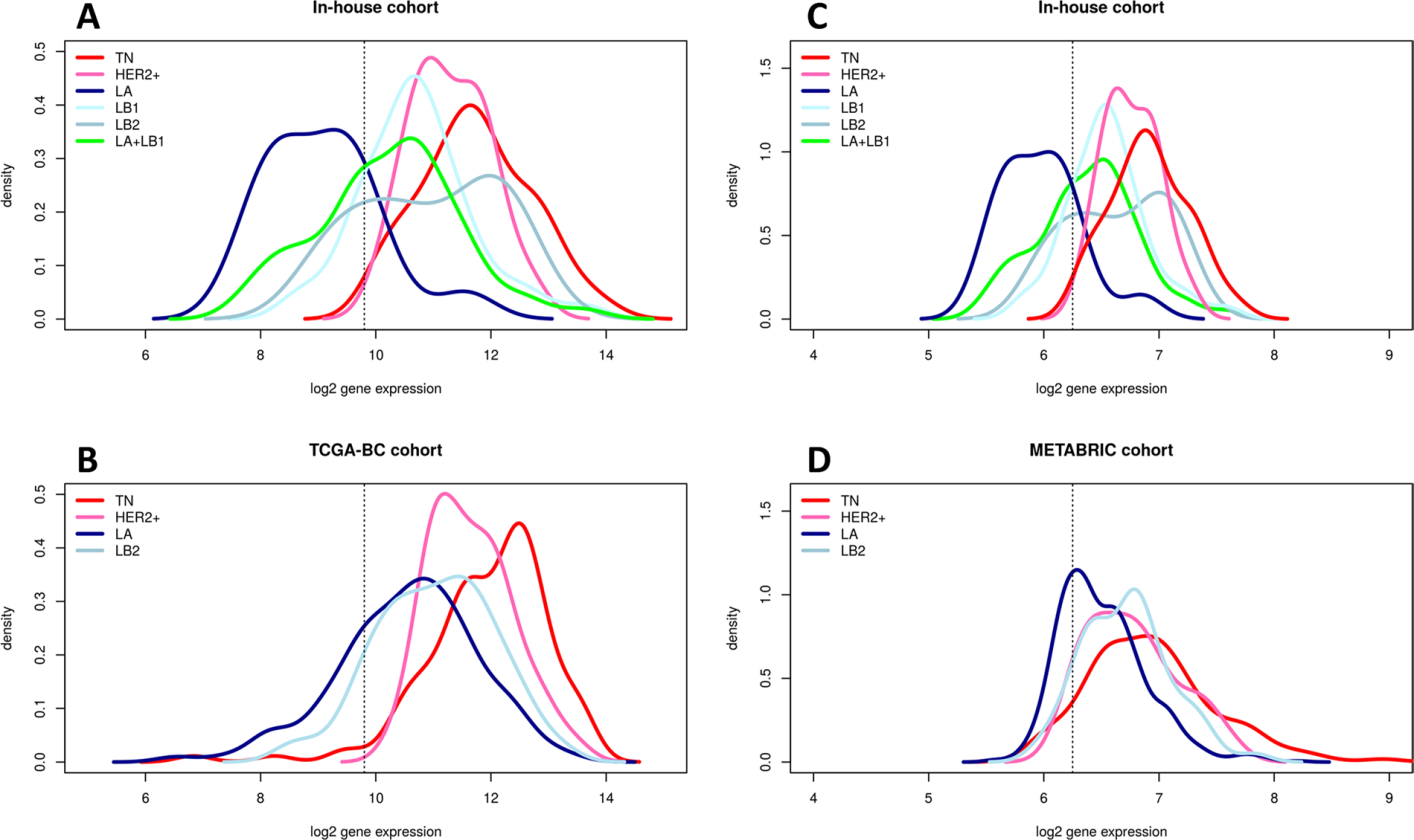
Density of *MKI67* gene expression among IHC subtypes in the studied datasets. The In-house cohort (**A**) was combined with TCGA (**B**) to facilitate comparison of data at the same scale (as described in Methods). The in-house cohort (**C**) was also combined with METABRIC (**D**) the same way. Vertical line is drawn at the cut-off where in-house data LA + LB1 density curve intersect LA & LB1 curves. This cutoff is applied to TCGA and METABRIC data to separate LA into LA & LB1.

Normalized gene expression data (upper-quartile) for 1097 primary breast tumors was retrieved from the TCGA data repository located at Genomic Data Commons [https://gdc.cancer.gov/], and this cohort is named TCGA-BC. Among these, a subset of 712 primary tumors with clinical IHC status available for ER, PR and HER2^24,33^. IHC subtypes were defined the same way as before for TN and HER2+, LA and LB2 subtypes. Since IHC status for Ki67 was not available to discriminate LB1 from LA (ER+/Her2−) cases, *MKI67* gene expression was used as a surrogate, given the positive correlation of *MKI67* gene and protein expression^34^. To decide the cutoff for *MKI67* we combined TCGA BC cohort and in-house cohort on common Ensembl gene identifiers after adjustment of the apparent batch effect using ComBat function in *sva*^35^ Bioconductor package. The combined matrix enabled the comparison of density distribution of *MKI67* between LA and LB1 of both in-house RNA-Seq and TCGA dataset on the same scale (Fig. 1A,B). The original scale for density distribution is provided as Supplemental Fig. S1A,B. To perform survival analysis we obtained TCGA BC survival data available for 1095 primary tumors from TCGA Clinical Data Resource^36^.

### METABRIC microarray data

Normalized gene expression data and clinical data of the discovery set were obtained from the METABRIC study^25^. Probes were converted to PAM50 genes as described in the paper. In the case of more than one high quality probe available for a gene, then the probe with highest variability was chosen (confirmed through personal communication). Two of the 50 genes were eliminated because of bad quality as described in the article. IHC subtypes were defined for 989 of the 997 cases the same way as before for TN, HER2+, LA and LB2 subtypes. As rationalized before, to separate LB1 from LA (ER+/HER2−) cases we combined METABRIC discovery set with in-house cohort on common 48 PAM50 genes and adjusted for batch effect. The combined batch-adjusted matrix enabled the comparison of density distribution of *MKI67* between LA and LB1 of both in-house and METABRIC cohort on the same scale (Fig. 1C,D). The density distribution in original scale is provided as Supplemental Fig. S1C.

### Statistical analysis

**Figure 2:**
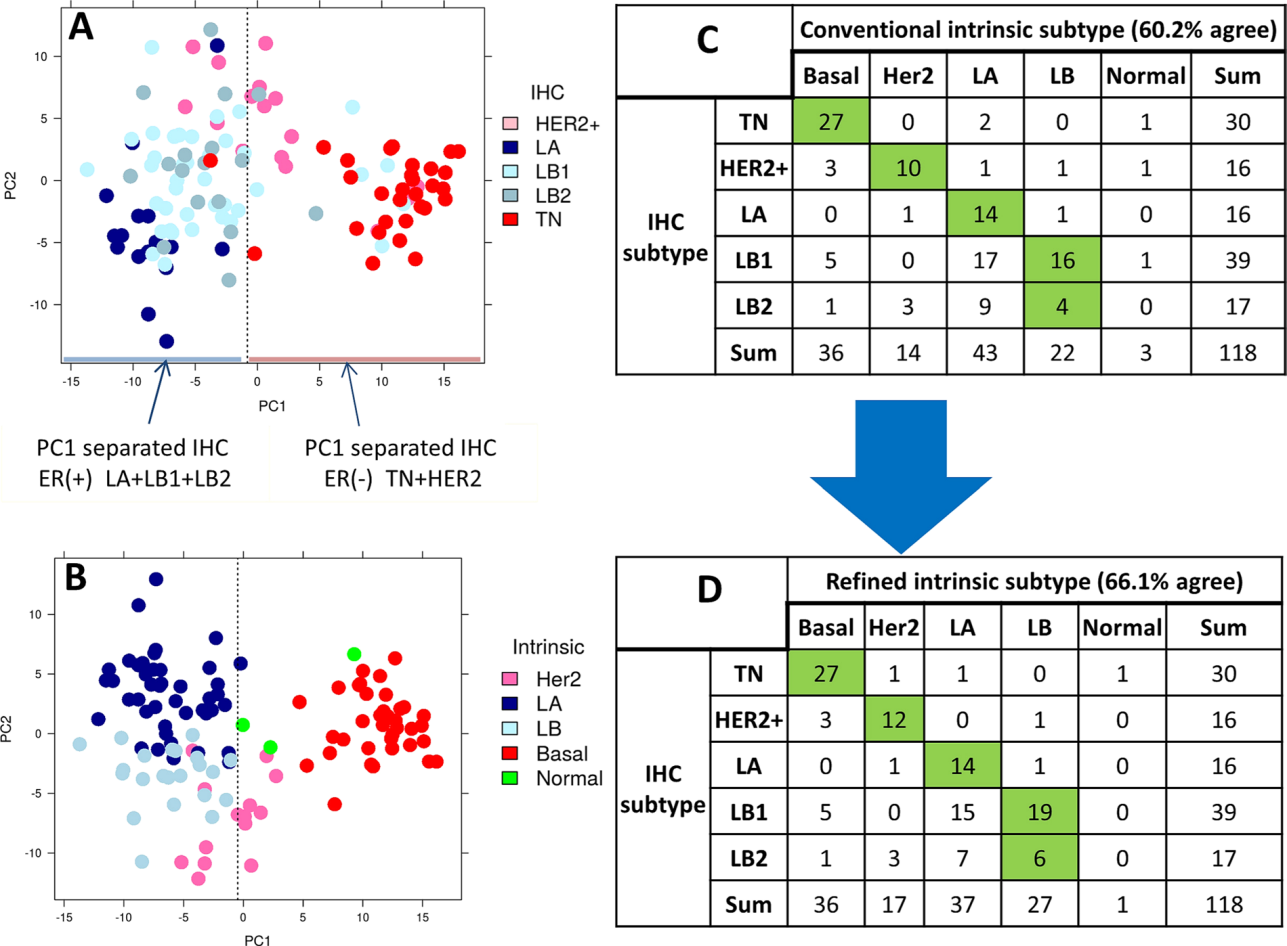
PCA plot and contingency table of in-house RNA-Seq dataset. (**A,B**) PCA plot of normalized PAM50 gene expression with IHC and intermediate intrinsic subtypes, respectively. (**C,D**) Contingency table comparing IHC subtypes to conventional and refined intrinsic subtypes, respectively.

**Figure 3:**
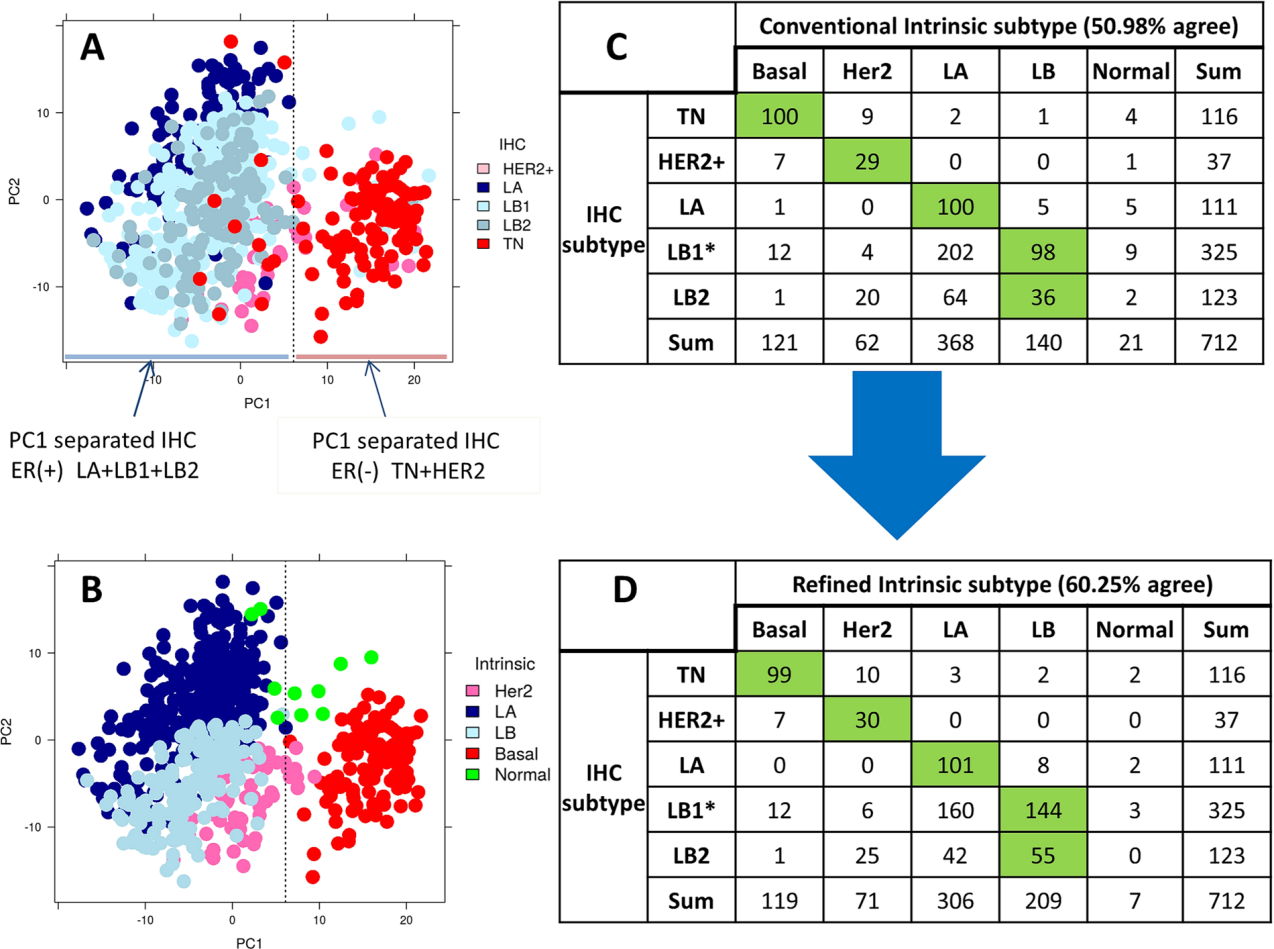
PCA plot and contingency table of TCGA RNA-Seq dataset. (**A,B**) PCA plot of normalized PAM50 gene expression with IHC and intermediate intrinsic subtypes, respectively. (**C,D**) Contingency table comparing IHC to conventional and refined intrinsic subtypes, respectively. *MKI67 gene expression was used to separate LB1 from LA.

**Figure 4:**
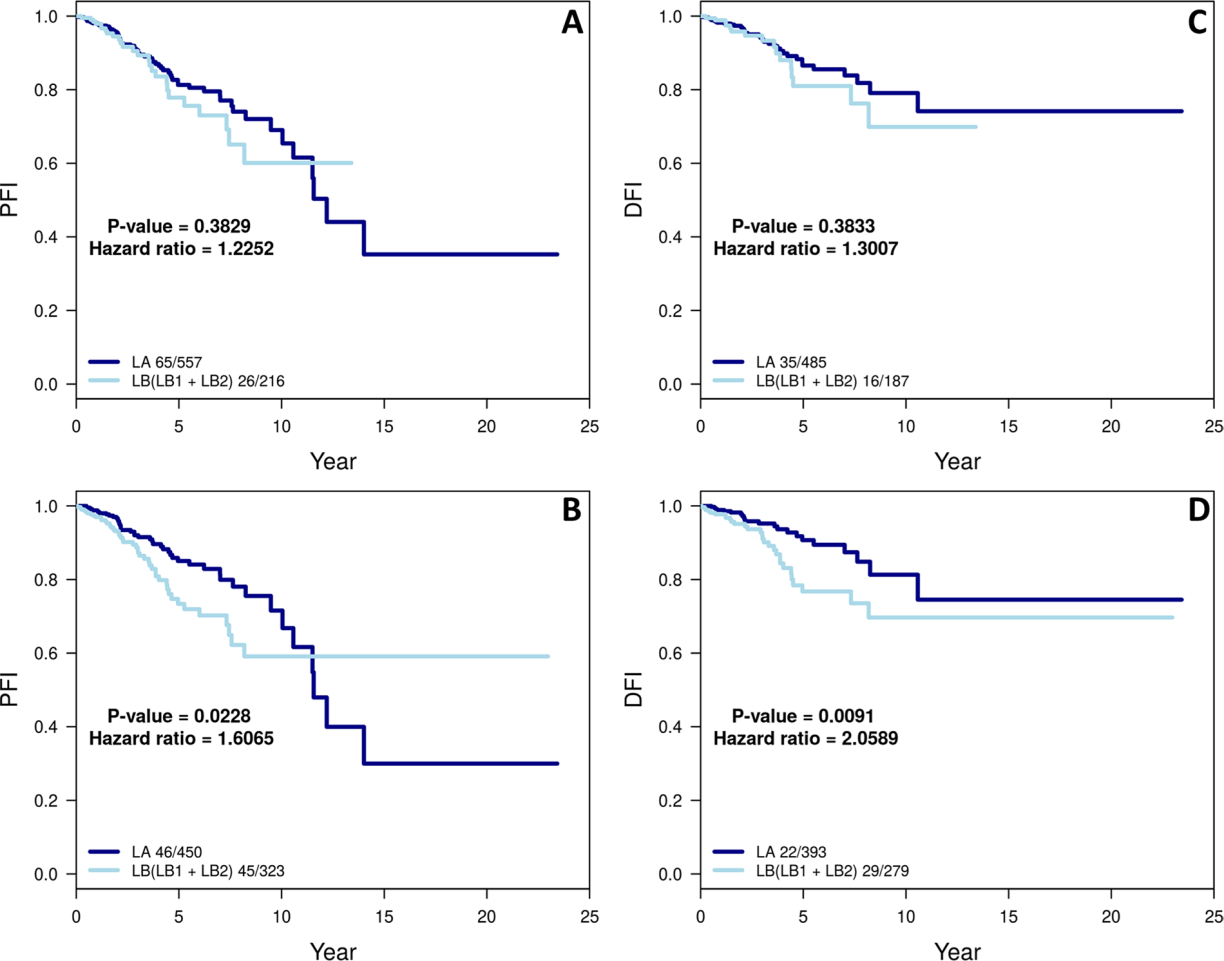
The Kaplan–Meier curves for cumulative survival in years for LA and LB cohorts as defined by conventional (**A,C**) and refined intrinsic subtypes (**B,D**) for two recommended end points in the TCGA BC cohort: progression-free interval (PFI) and disease-free Interval (DFI). P-value and the number of events ‘/’ number of cases are given in the legends of plots.

PCA and hierarchical clustering were performed using Bioconductor packages *Lattice*^37^, *Genefilter*^38^ and *gplots* R/Bioconductor package version 3.4.0^39^. PCA was performed using PAM50 genes. Wilcoxon rank sum test was used for statistical significance (p < 0.05). Survival analyses were performed using the *survival* Bioconductor package^40^. Log rank test was used to test difference in survival between groups. Cox proportional hazard regression was used to obtain hazard ratio. To decide the cutoff on the PCA map to separate ER negative and positive cases, we calculated misclassified cases at each point on PC1 axis. The point on PC1 axis with minimum percentage of misclassified cases was identified as the cutoff. Our assumption for the following formula was that the majority of ER positive and negative cases are on the PC1 negative and positive axis respectively (Figs 2A, 3A and 4A). In order to satisfy our assumption, the input data was arranged in such a way that all or most of the ER-positive cases appear in the beginning of the matrix. For a specific point x on PC1, the percentage of misclassified cases (Px) was defined as,

$$Px=(\frac{Rx}{R}+\frac{Nx}{N})\,\ast \,100$$

where Rx is the number of IHC ER-positive (LA + LB1 + LB2) cases greater than or equal to given point x, R is the total number of IHC ER-positive cases. Nx is the number of IHC ER-negative (TN + HER2+) cases less than the given point x, N is the total number of IHC ER-negative cases. Supplemental Fig. S2A,B, and C provide the percentage of misclassified classes across PC1 axis for in-house, TCGA 712-case and METABRIC 989-case cohorts respectively.

### Intrinsic subtyping using conventional PAM50 and new PCA-PAM50

PAM50 classifier^5^ was used to call intrinsic subtypes. Gene centering of the normalized PAM50 gene expression matrix was conducted using median values obtained from the ER-balanced subset. It is important to note that gene expression was normalized using upper quartile as used previously to be compatible with PAM50 centroid^5,20,41^. Three different ER-balanced (50% ER−, 50% ER+) subsets were derived. Since there were more ER-positive cases in the datasets compared to ER-negative cases, an ER-balanced subset was chosen by randomly selecting an equal number of ER-positive cases to match ER-negative cases. The primary ER-balanced set was selected based on IHC status: ER-negative cases = all of TN and Her2+ cases; ER-positive cases = same number as the ER-negative cases but randomly selected from LA, LB1, and LB2 subtypes. The PAM50 calls resulting from the use of the primary ER-balanced subset are called conventional intrinsic subtypes. The secondary ER-balanced set was based on principal component 1 (PC1) separation that agreed with IHC status: ER-negative cases = TN and Her2+ cases that were on the right side of the PC1 cutoff; ER-positive cases = same number as the ER-negative cases but randomly selected from LA, LB1, and LB2 present on the left side of the PC1 cutoff (Figs 2A, 3A and 4A). The PAM50 calls resulting from the use of the secondary ER-balanced subset are called intermediate intrinsic subtype. The tertiary ER-balanced set was based on the intermediate intrinsic subtype’s Basal and LA calls: ER-negative cases = all of the Basal cases; ER-positive cases = same number as the ER-negative cases but randomly selected from LA cases. The PAM50 calls resulting from the use of the tertiary ER-balanced set are called refined intrinsic subtypes. The new method we proposed here is named as PCA-PAM50 to distinguish from the convention PAM50. For the TCGA and METABRIC datasets, the provided PAM50 calls were considered as conventional intrinsic subtype.

### Ethics approval and consent to participate

As mentioned elsewhere in previous CBCP publication^42^ in house data collection was conducted in accordance with a minimal risk protocol entitled “Tissue and Blood Library Establishment for Molecular, Biochemical and Histologic Study of Breast Disease”, approved by the IRB of the Walter Reed National Military Medical Center (IRBNet #20704) for the CBCP. Written informed consent was given by participants for use of their medical records and additional data collected through the CBCP questionnaires and data forms. The consent also allowed biospecimen procurement and subsequent use for genomic and proteomic experiments. Research using these molecular and clinicopathologic data was covered by the protocol, including this specific study reported here as part of the CBCP. We followed the proper guidelines to obtain publicly available TCGA^24^ and METABRIC^25^ data (Methods).

### Disclosure of prior publication

These analyses were presented previously in part in abstract form^43^.

## Results

### Evaluation of PCA-PAM50 method using In-house cohort

We use PCA, a multivariate technique that does orthogonal transformation, to reduce high dimensional data into a number of components to display data similarity as points on maps. This transformation is defined in such a way that the first principal component (PC1) has the largest possible variance. In our in-house RNA-Seq dataset, the PCA map grouping of cases overlaid with IHC subtypes indicated that the PC1 parted most, but not all, of the ER- positive (LA + LB1 + LB2) and ER-negative (TN and Her2+) cases (Fig. 2A). This motivated us to consider removing the inconsistency between the IHC-based and gene expression-based ER status in the conventional method of selecting an ER-balanced subset (primary ER-balanced set; see Methods). Therefore, we proceeded to derive gene expression-based ER status using three steps, which were as follows: The first step was to identify ER-positive and ER-negative cases that are separated on PC1 axis. To form cutoff on PC1 we calculated the percentage of misclassified cases at every point on PC1 (−10 to 10; Supplemental Fig. S2A) and used the point with minimum percentage of misclassified cases as PC1 cut-off, which was −0.81. As verification we also observed the *ESR1* gene expression was significantly (p value = 3.2 × 10^−17^) different across the PC1 cutoff (Supplemental Fig. S3A,B). This led us to form a secondary ER-balanced subset based on both PC1 separation and IHC subtype (Fig. 2A). The second step was to do a first iteration of PAM50 using the secondary ER-balanced subset resulting in an intermediate intrinsic subtype. This subtype on the PCA map distinguished Basal and LA as two well-separated components (Fig. 2B). This led us to define a gene-expression based tertiary ER-balanced subset. The final step uses the tertiary ER-balanced subset to make a final PAM50 classification, which we refer to as a refined intrinsic subtype. These three steps form our PCA-PAM50 method.

As defined in the methods, PAM50 calls resulting from gene centering procedures using primary ER-balanced set are called conventional intrinsic subtypes. Both the conventional and refined intrinsic subtypes were compared to the clinical subtypes (Fig. 2C,D). The agreement among the intrinsic and clinical subtyping improved with refined labels by approximately 6%. Especially, the LB calls have increased agreement from 20 to 25 cases (a 25% increase) with the total increase in LB calls from 22 to 27 (Fig. 2C,D). Even though LA intrinsic calls decreased from 43 to 37 in the refined calls, their agreement with the IHC subtype remained the same (n = 14). In addition to LB, Her2 also increased in consistency from 10 to 12 cases (a 20% increase). Notably, the normal-like intrinsic call was decreased from 3 to 1 in refined subtype. However, in this dataset the numbers of changed cases are small. Hence, we proceeded to evaluate PCA-PAM50 method on publically available larger cohorts.

### Evaluation of PCA-PAM50 method using TCGA-BC cohort

For subtyping consistency analysis, we focused on the 712 TCGA-BC cases that had IHC status available. IHC subtypes were defined for TN, HER2+, LA, LB1 and LB2 as described earlier. Similar to the in-house dataset, the 712 TCGA-BC data PCA map grouping of cases also does not perfectly reflect the ER-status derived from IHC subtypes (Fig. 3A). The PC1 cut-off was calculated as defined earlier (Supplemental Fig. S2B). Furthermore, *ESR1* gene expression across PC1 cutoff (PC1 = 6.1) was very similar (p value = 2.7 × 10^−65^) to that described for the in-house cohort (Supplemental Fig. S3C,D). Therefore, we employed our three step PCA-PAM50 method to develop a secondary and tertiary ER-balanced subset to derive the refined intrinsic subtype. Figure 3B is a PCA map overlaid with the intermediate intrinsic subtype from which we formed the tertiary ER-balanced subset. The intrinsic subtypes provided along with the data resource were referred to as the conventional intrinsic subtype. Among the 712 cases, the consistency between the conventional intrinsic subtype and the IHC subtype was 50.98% (Fig. 3C). When using the refined intrinsic subtype, this consistency increased to 60.25% (Fig. 3D; a 9.3% increase). Again, the consistency of intrinsic LB compared to IHC LB increased from 134 to 199 (~49% increase). The total LB calls increased from 140 in the conventional intrinsic to 209 in the refined intrinsic (Fig. 3C,D). Even though the number of LA calls decreased from 368 to 306, the consistency with IHC LA did not decrease. Notably, normal-like subtype decreased from 21 in the conventional intrinsic to 7 in the refined intrinsic subtype (Fig. 3C,D).

Next we applied the median values derived from tertiary ER-balanced subset of the 712-case cohort to gene center the entire TCGA BC’s 1097-case cohort to obtain refined intrinsic subtype. Table 1 gives the comparison of conventional (provided) with refined intrinsic subtype for the 1097-case cohort. To assess clinical significance of these subtype calls, we performed a Kaplan-Meier analysis for patients with LA and LB subtypes of tumors called by the conventional and refined intrinsic subtyping (Table 1; cases underlined, and one patient did not have survival data available). The two recommended clinical outcome endpoints were used^36^: progression-free interval (PFI), and disease-free interval (DFI). For both end points, the survival outcome difference became significant (p value = 0.0228 for PFI and 0.0091 for DFI; Fig. 4B,D) when comparing refined intrinsic to conventional intrinsic (p value = 0.3829 for PFI and 0.3833 for DFI; Fig. 4A,C). We also observed a significantly (p value = 4.11 × 10^−21^) higher *MKI67* gene expression for the 107 cases that switched from LA in conventional intrinsic to LB in refined intrinsic subtype (Supplemental Fig. S4B) for the TCGA BC cohort (Table 1). This is comparable to the significantly (p value = 0.00124) higher *MKI*67 gene expression for the 5 cases that switched from LA in conventional intrinsic to LB in refined intrinsic subtype (Supplemental Fig. S4A) for the In-house BC cohort (Supplemental Table S1).

**Table 1:** Contingency table comparing conventional and refined intrinsic subtypes among 1097-case TCGA BC cohort.

		Refined intrinsic subtype					
		Basal	Her2	LA	LB	Normal	Sum
**Conventional** **Intrinsic** **subtype**	**Basal**	185	5	0	2	0	192
	**Her2**	0	82	0	0	0	82
	**LA**	0	7	452	107	0	566
	**LB**	0	1	0	216	0	217
	**Normal**	1	5	21	2	11	40
	**Sum**	186	100	473	327	11	1097

For the plots in Fig. 4, we used the patients that are either LA or LB in both conventional and refined subtypes (underlined).

### Evaluation of PCA-PAM50 method using METABRIC discovery set

**Figure 5:**
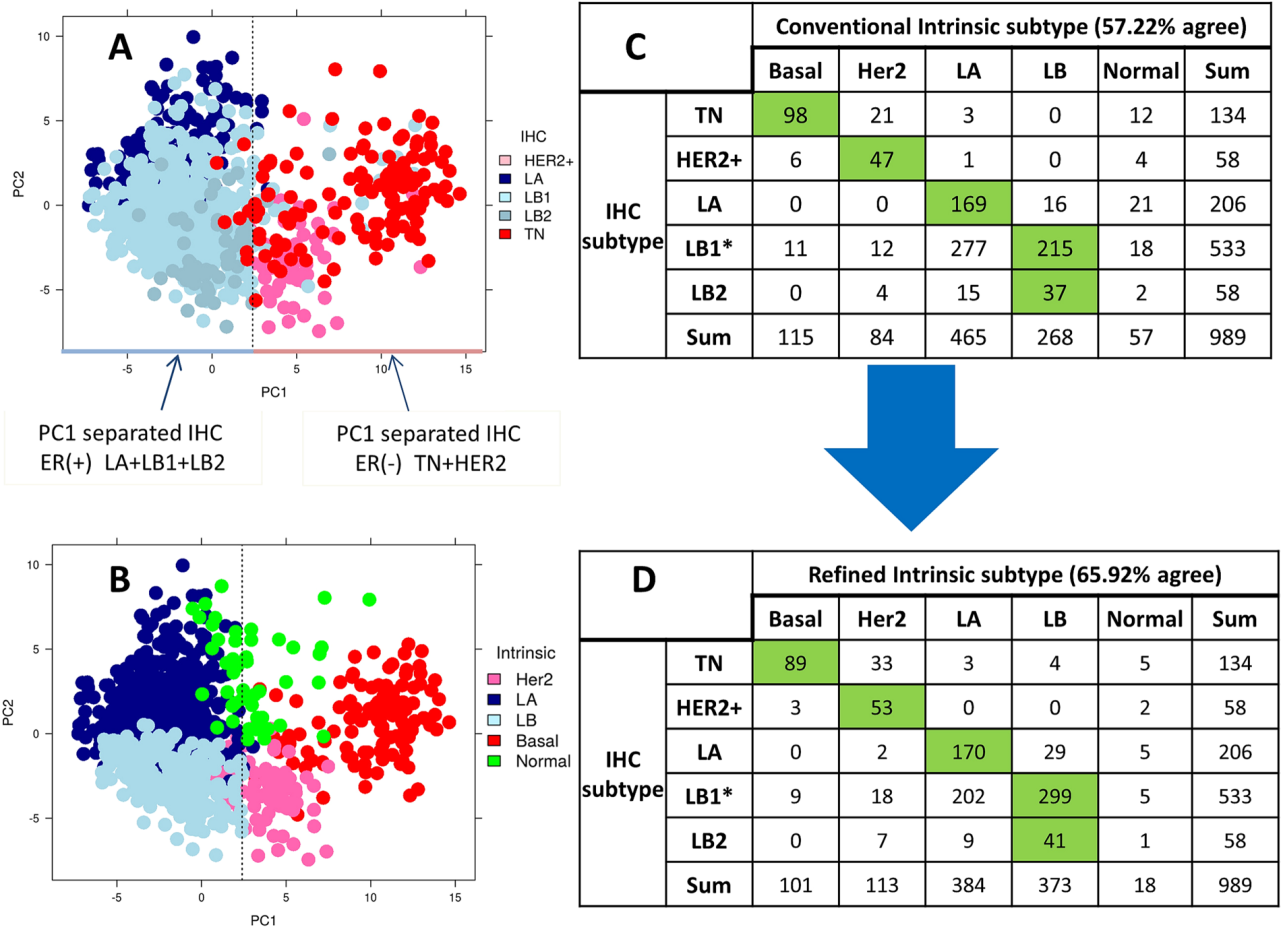
PCA plot and contingency table of METABRIC discovery set. (**A,B**) PCA plot of normalized PAM50 gene expression with IHC and intermediate intrinsic subtypes, respectively. (**C,D**) Contingency table comparing IHC to conventional and refined intrinsic subtypes, respectively. *MKI67 gene expression was used to separate LB1 from LA.

Here we employed our PCA-PAM50 method on the 989-case METABRIC discovery set for which we deduced IHC subtypes. Similar to in-house and TCGA dataset, the METABRIC data PCA map grouping of cases also does not perfectly reflect the ER-status derived from IHC subtypes (Fig. 5A). The PC1 cut-off was calculated as defined earlier (Supplemental Fig. S2C). Furthermore, *ESR1* gene expression across PC1 cutoff (PC1 = 2.4) was significantly (p value = 2.06 × 10^−104^) different, which is similar to the in-house and TCGA cohorts (Supplemental Fig. S3E,F). Using the PCA-PAM50 method, we developed secondary and tertiary ER-balanced subsets to obtain the refined intrinsic subtype. Figure 5B is a PCA map overlaid with intermediate intrinsic subtypes with which the tertiary ER-balanced subset is formed. Among the 989 cases, the consistency between conventional intrinsic subtype (provided along with data source) and the IHC subtype was 57.22% (Fig. 5C). This consistency increased to 65.92% with refined intrinsic subtype (an 8.7% increase; Fig. 5D). Again, the consistency of LB with IHC LB increased from 252 to 340 (a 35% increase), with the total LB calls increasing from 268 to 373 (Fig. 5C,D). Even though the number of LA calls decreased from 465 to 384, the consistency with IHC LA did not decrease. Notably, normal-like subtype decreased from 57 in the conventional intrinsic to 18 in the refined intrinsic (Fig. 5C,D).

**Figure 6:**
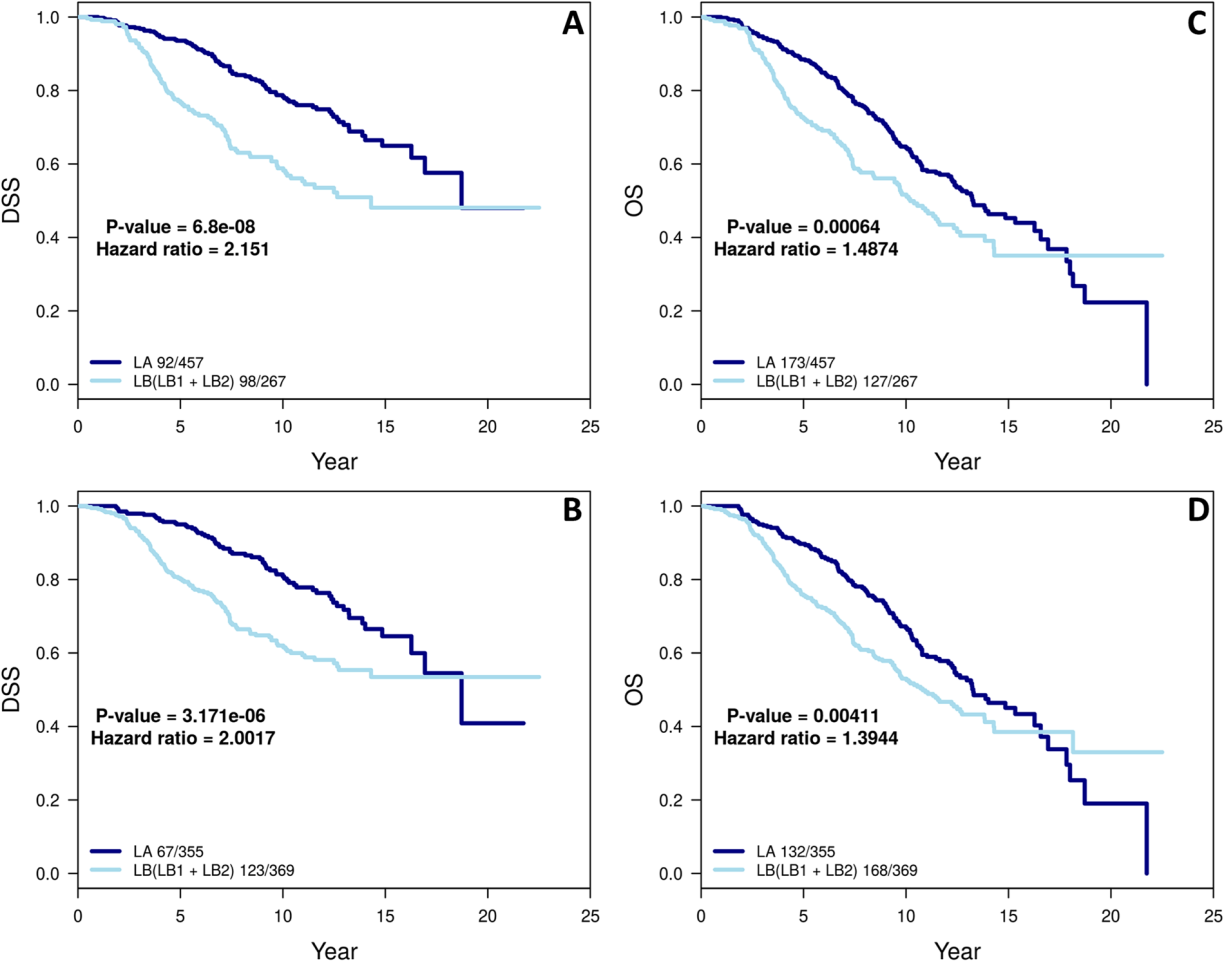
The Kaplan–Meier curves for cumulative survival in years for LA and LB cohorts as defined by conventional (**A,C**) and refined intrinsic subtypes (**B,D**) for all two available end points in METABRIC cohort: disease-specific survival (DSS) and overall survival (OS). P-value and the number of events ‘/’ number of cases are given in the legends of plots.

Table 2 shows the comparison of conventional (provided) with refined intrinsic subtypes. To examine whether the change in subtype calls for patients from LA to LB is reflected in survival differences, we performed a Kaplan-Meier analysis for patients with these two subtypes of tumors called by the conventional and refined intrinsic subtyping (Table 2; cases underlined). For METBARIC cohort only two end points are available: overall survival (OS) and disease specific survival (DSS). For both these end points the refined classifications of LA and LB remain significantly different in survival outcomes (Fig. 6). Similar to in-house and TCGA cohorts we observed that there was significantly (p value = 2.3 × 10^−13^) higher *MKI67* gene expression for the 102 cases that switched from LA to LB between conventional and refined intrinsic subtype (Supplemental Fig. S4C; Table 2).

**Table 2:** Contingency table comparing conventional and refined intrinsic subtypes among all 989-case METABRIC study cohort.

		Refined intrinsic subtype					
		Basal	Her2	LA	LB	Normal	Sum
**Conventional** **Intrinsic** **subtype**	**Basal**	100	13	0	2	0	115
	**Her2**	0	84	0	0	0	84
	**LA**	0	8	355	102	0	465
	**LB**	0	1	0	267	0	268
	**Normal**	1	7	29	2	18	57
	**Sum**	101	113	384	373	18	989

For the plots in Fig. 6, we used the patients that are either LA or LB in both conventional and refined subtypes (underlined).

### Survival analysis for switched cases

**Figure 7:**
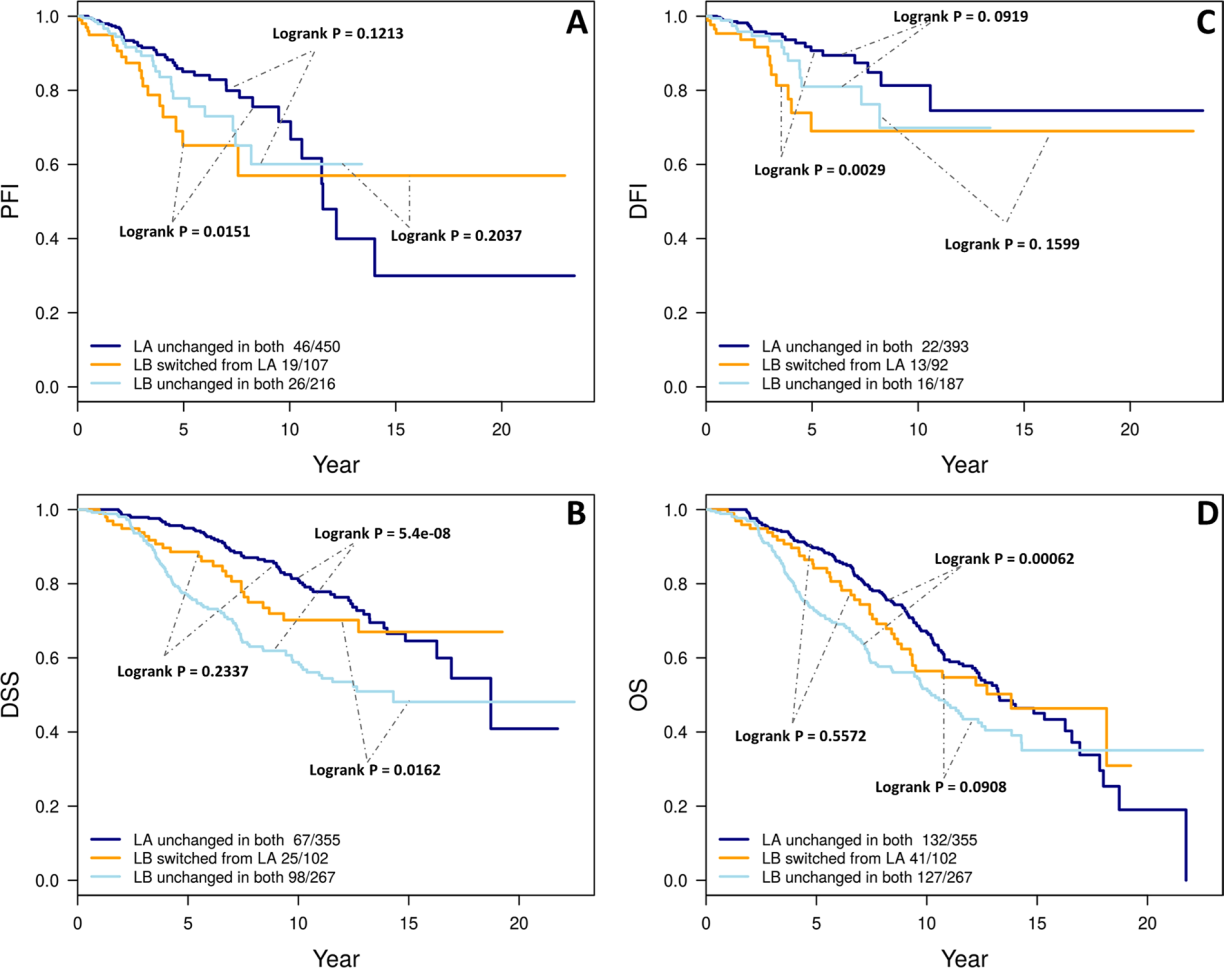
Difference in survival among the cases that remained as LA and LB in both conventional and intrinsic subtype and the cases switched from LA in conventional to LB in refined subtype for both TCGA (**A,C**) and METABRIC (**B,D**) cohorts. For the TCGA-BC cohort, two recommended^36^ end points are used: progression-free interval (PFI) and disease-free Interval (DFI). For METABRIC cohort, both available end points are used: disease-specific survival (DSS) and overall survival (OS). P-value and the number of events ‘/’ number of cases are given in the legends of plots.

To examine if the patients that switched from LA to LB between conventional and refined intrinsic subtype have any clinical implication, we performed Kaplan-Meier survival analyses using the TCGA-BC and METABRIC cohorts patients that fall into three different categories: (1) those that remained LA in both conventional and refined intrinsic subtype, (2) those that switched from conventional LA to LB in refined intrinsic subtype, (3) those that remained LB in both conventional and refined intrinsic subtype (Tables 1 and 2). For TCGA-BC cohort, we used two recommended clinical outcome endpoints^36^: progression-free interval (PFI), and disease-free interval (DFI). We found that the cases switched from LA to LB showed a significant survival difference from cases that remained unchanged as LA for both end points (p value = 0.0151 for PFI and 0.0029 for DFI; Fig. 7A,C), and a trended worse survival outcome from those remained unchanged as LB (p value = 0.2037 for PFI and 0.1599 for DFI; Fig. 7A,C). For METABRIC cohort, the two available end points are used: overall survival (OS) and disease specific survival (DSS). Here we observed a trended worse survival in the cases that switched from LA to LB compared to those remained unchanged as LA in DSS (p value = 0.2337, Fig. 7B) although the difference was not significant in OS (p value = 0.5572, Fig. 7D). Yet we observed significant or strong-trended survival difference between the cases switched from LA to LB and cases that remained unchanged LB (p value = 0.0162 for DSS and 0.0908 for OS). It is important to note that the PAM50 subtyping procedure used in METABRIC was different from conventional one and it involves 100 trials from which consensus calls were derived to be known as “conventional” PAM50 intrinsic subtype calls. In this cohort patients with “conventional” intrinsic LB subtype tumors had already demonstrated significantly worse clinical outcomes compared to those with LA tumors. We will revisit this in the Discussion section.

## Discussion

The heterogeneous nature of breast cancer is defined by four main intrinsic subtypes (LA, LB, Her2 and Basal)^1,5^. In the clinical setting, IHC subtyping for these four classifications uses 3–4 biomarkers which forms the basis for patient treatment decision-making^13–17^. The marker Ki67 is used in many institutions to derive comparable IHC LA, LB to its Intrinsic counterpart^9–12^. However, the agreement between Intrinsic and IHC subtype is often reported to be around 75% or lower for Basal/TN, and much lower for other subtypes^18,44^. Here, we present a method termed PCA-PAM50 to choose a gene expression-guided, ER-balanced subset yielding a refined intrinsic subtyping. Our method resulted in improved consistency between refined intrinsic and IHC subtypes than that of conventional intrinsic subtypes for all three datasets: in-house, TCGA BC, and METABRIC cohorts. The significant increase in consistency is due to the increase in intrinsic LB calls agreeing with IHC’s LB by 25–49% (Figs 2, 3 and 5). The next best increase in consistency was with Her2 calls by 3.4–12.7%. Even though the number of LA calls decreased, the consistency with IHC LA did not decrease. The consistency of Basal with IHC’s TN decreased by 0% (in-house), 1% (TCGA BC), and 9% (METABRIC) respectively. For the latter (Fig. 5C,D), the PCA map separation seems to suggest refined intrinsic subtype may be more accurate as all of the Basal subtype are grouped to the right side of the PC1 cutoff (Supplemental Fig. S5) unlike with the IHC subtype where TN is also present on the left side of the PC1 cutoff (Fig. 5A). We understand that before the current 1% cut-off for ER IHC assay was established^32^, different clinical sites exercised different cut-offs ranging from 5% to 20%^13,45^ thus previous ER-negative cases could become ER-positive by the current standard, which may explain some of the TN cases presenting on the left (ER+) side of the PC1 cutoff. We found that the 9 TN cases (=98–89) that are no longer consistent with the IHC TN subtype are now classified as LB (n = 2) and Her2 (n = 7) in PCA-PAM50 subtyping. Taken together, PCA-PAM50 yielded higher consistency with IHC subtypes for LB and HER2+, comparable consistency for LA, and slightly decreased consistency with the TN subtype which may be partially attributed to the higher thresholds for ER+ due to the historic evolution of the standards for this marker.

LB tumors are regarded as more aggressive than LA tumors due to the expression of Her2 and Ki67 which are often associated with a higher proliferation rate^46–48^. Ki67 is a nuclear protein associated with cellular proliferation, a well-established marker for separating LB from LA apart from the Her2 status^12,16,49,50^. Multiple studies have shown that high expression of Ki67 protein is associated with poor prognosis^51–54^, and therefore, we expected the *MKI67* gene, a proliferation marker associated with poor prognosis of LB patients, to be high in cases that switched from LA to LB between PAM50 calls. As expected, we observed that cases that switched from LA to LB did show significantly higher *MKI67* gene expression than cases that remained LA in both refined and conventional subtypes (Supplemental Fig. S4). There may be a concern that it might be because we used *MKI*67 gene expression as a surrogate for Ki67 protein expression to distinguish IHC subtypes LB1 from LA. However, it does not have any influence in the PCA-PAM50 method because it does not impact the choice of the ER-balanced set. The LB1 separation from LA makes the TCGA and METABRIC’s IHC subtypes comparable to that of the in-house cohort (Figs 2C,D, 3C,D and 5C,D).

The conventional PAM50 subtyping procedure is very important and provided a valuable intrinsic subtype to the breast cancer research community. However, it is often the case that PAM50 subtypes do not fully capture the difference in luminal tumors as done by clinically defined biomarkers^8,55^. In the TCGA-BC cohort where the standard conventional PAM50 subtyping method was used, patients with conventional LB did not demonstrate significantly worse clinical outcomes compared to those with the LA subtype which is likely due to the relatively short clinical follow-up time. Our new method, PCA-PAM50, reclassified 107 conventional LA tumors as LB (Table 1). This subset of patients demonstrated significantly worse survival outcomes compared to the LA cases that remained unchanged (Fig. 7). It is this subset of patients that resulted in ultimate difference in clinical outcomes between refined LA and LB in the TCGA-BC cohort (Fig. 4B,D). This is clinically important. Taken together with improved consistency to the IHC LB subtype (Fig. 3D) and the higher expression of the *MKI67* gene in this switched cases (Supplemental Fig. S4), we conclude that PCA-PAM50 identified a clinically important subset of tumors that probably should be subtyped as LB instead of LA.

In the METABRIC cohort, the results appeared to be not as striking since the cases switched from LA to LB only demonstrated a trended worse DSS compared to those remained as LA, and almost no difference in OS; note that it is the DSS that is more important an outcome endpoint in breast cancer studies especially for patients with luminal subtypes of tumors since those patients are often diagnosed at an older age, and that deaths due to comorbidities in older patients are more profound to mask the deaths due to the disease in survival analysis. From the algorithm perspective, PAM50 subtyping used in METABRIC^25^ is unconventional and it involves 100 subsets with 100 trials and the final “conventional” subtype calls were derived as consensus from the 100 trials. This consensus “conventional” PAM50 subtyping may have resulted in more stable intrinsic subtype calls, and this may be one contributing factor that the switched cases identified by PCA-PAM50 did not demonstrate worse clinical outcomes as strongly as in the TCGA-BC cohort where original subtypes were really derived from conventional PAM50 subtyping. We also note that the METABRIC dataset has a longer clinical follow-up time compared to the TCGA-BC dataset.

We show that for TCGA-BC cases, when the refined intrinsic subtypes became more consistent with the IHC subtypes, refined LB cases demonstrated significantly worse PFI and DFI compared to refined LA cases (Fig. 4B,D). This brought up a question whether clinical IHC subtyping actually is still a stronger prognosis predictor than PCA-PAM50 subtyping. To examine this possibility we performed Kaplan-Meier analysis for patients with LA and LB IHC subtypes among the data used for Fig. 4. In the TCGA-BC cohort, only 502 cases out of 774 cases used in Fig. 4 had IHC data. Among these 502 cases, there were 105 LA, 394 LB (LB1 + LB2) and 3 TN. Unfortunately, the number of events is too low to draw any conclusions (Supplemental Fig. S6A,B). Turning to the METABRIC cohort, all 724 cases used in Fig. 6 had IHC data. Among these 724 cases, there were 184 LA, 539 LB (LB1 + LB2) and 1 TN. Please note *MKI67* gene expression was used to separate LB1 from LA (Fig. 1). The DSS difference was significant for IHC subtypes (p value = 5e-04, HR = 1.98, Supplemental Fig. S6B) and it is similar to the difference with refined intrinsic subtypes (p value = 3e-6, HR = 2.00, Fig. 6). For OS, the difference in IHC subtypes is also significant but with a higher HR (p = 0.0012, HR = 1.61) compared to the difference with the refined intrinsic subtypes (p value = 0.0041, HR = 1.39). However, as discussed earlier, OS is less reliable an endpoint compared to DSS for luminal disease survival analysis. Thus our conclusion is that, IHC subtyping may be comparable a prognosis predictor to our new PCA-PAM50 subtyping.

We also compared the IHC consistency of the METABRIC refined intrinsic subtype with that of the updated/refined intrinsic subtypes of Milioli *et al*.^19^. The authors Milioli *et al*., presented the updated intrinsic subtypes for the cohort which is claimed to be more consistent with the clinical/IHC subtypes than the original report^25^. However, the overall agreement and specifically, the LB agreement, is greater with PCA-PAM50 for the METABRIC cohort than both Milioli *et al*., and the original report (compare Supplemental Table S2 and Fig. 5C,D). We also note that the PCA-PAM50 method is designed to perform intrinsic subtyping in an ER-status unbalanced study cohort. As such, it requires the input cohort to have both ER-negative and ER-positive cases in some proportion. For datasets with only one type of ER-status, users are advised to assess other available methods, for example^56^.

One unexpected result from this study is the decrease in the number of normal-like subtype calls from 40 and 57 in conventional intrinsic subtyping to 11 and 18 in refined intrinsic subtyping in the TCGA and METABRIC cohorts respectively (Tables 1 and 2). Clinical insight into this decreased normal-like subtype call will be a worthy project given that the normal-like subtype is suggested as a result of contamination with normal breast tissue^6–8^. Furthermore in the original PAM50 classifier paper^5^ it was articulated that normal-like centroid was developed by training on normal breast tissue. Another observation is that although the number of LA calls was largely reduced from conventional intrinsic subtyping to refined intrinsic subtyping, the cases that were consistent with IHC subtyping were not reduced. Again this observation warrants additional studies to identify the underlying reasons that may be of clinical value.

## Conclusion

In conclusion, we have developed a new breast cancer subtyping method called PCA-PAM50, which couples the conventional PAM50 method with PCA for selection of a gene expression–based ER-balanced subset as the first step for intrinsic subtyping. The new method enhances the consistency between intrinsic and clinical subtype calls, which is especially observed in the LB subtype that bears the highest number of clinical breast cancer recurrences. The new LB subtyping was supported by *MKI67* and survival outcome analyses. Two other noteworthy findings, that the reduced new LA subtype calls did not cause a reduction in the consistent LA calls, and that normal-like subtype calls were drastically reduced, are potentially clinically important and warrant additional studies. This study provides an additional method for breast cancer subtyping which may be proven valuable to mend the gap between clinical and research subtyping of breast cancers. The identification of a more aggressive subset from the conventional intrinsic LA subtype cases as LB may prove to be clinically important.

## Data Availability

TCGA breast cancer RNA-Seq dataset is available in the Genomic Data Commons repository (https://gdc.cancer.gov/) after acquiring permission to access restricted data. METABRIC molecular and clinical data are available from the original publication^25^ but again restrictions apply to the availability of the data. In-house RNA-Seq data were part of a proteogenomic study in preparation^57^ which will be published with that study. The PCA-PAM50 code for deriving the refined intrinsic subtype is available at ftp://ftp.wriwindber.org/.

## Supplementary information

Supplemental Tables and Figures
