# Supplementary material for: PCA-PAM50 improves consistency between breast cancer intrinsic and clinical subtyping reclassifying a subset of luminal A tumors as luminal B
Source: Sci Rep. 2019 May 28;9:7956. doi: 10.1038/s41598-019-44339-4 (PMC6538748; doi:10.1038/s41598-019-44339-4)

PCA-PAM50 improves consistency between breast cancer intrinsic and clinical subtyping reclassifying a subset of luminal A tumors as luminal B

***Praveen-Kumar Raj-Kumar^1^, Jianfang Liu^1^,*** ***Jeffrey A. Hooke^2^, Albert J. Kovatich^2^, Leonid Kvecher^1^, Craig D. Shriver^3^, and Hai Hu^1*^***

^1^Chan Soon-Shiong Institute of Molecular Medicine at Windber, Windber, PA.

^2^Clinical Breast Care Project, Murtha Cancer Center Research Program, Uniformed Services University of Health Sciences / Walter Reed National Military Medical Center, Bethesda, MD.

^3^Murtha Cancer Center Research Program, Uniformed Services University of Health Sciences / Walter Reed National Military Medical Center, Bethesda, MD.

*Corresponding author email: H.Hu@wriwindber.org

Table of Contents

[Supplemental Tables 3](#_Toc6227226)

[Supplemental Table S1 3](#_Toc6227227)

[Supplemental Table S2 4](#_Toc6227228)

[Supplemental figures 5](#_Toc6227229)

[Supplemental Figure S1 5](#_Toc6227230)

[Supplemental Figure S2 6](#_Toc6227231)

[Supplemental Figure S3 7](#_Toc6227232)

[Supplemental Figure S4 8](#_Toc6227233)

[Supplemental Figure S5 9](#_Toc6227234)

[Supplemental Figure S6 10](#_Toc6227235)

## Supplemental Tables

Supplemental Table S1**:** Contingency table comparing conventional and refined intrinsic subtypes among 118 In-house cohort.

|  | | **Refined intrinsic subtype** | | | | | |
| --- | --- | --- | --- | --- | --- | --- | --- |
|  |  | **Basal** | **Her2** | **LA** | **LB** | **Normal** | **Sum** |
| **Conventional**  **Intrinsic**  **subtype** | **Basal** | 36 | 0 | 0 | 0 | 0 | 36 |
|  | **Her2** | 0 | 14 | 0 | 0 | 0 | 14 |
|  | **LA** | 0 | 2 | 36 | 5 | 0 | 43 |
|  | **LB** | 0 | 0 | 0 | 22 | 0 | 22 |
|  | **Normal** | 0 | 1 | 1 | 0 | 1 | 3 |
|  | **Sum** | 36 | 17 | 37 | 27 | 1 | 118 |

Supplemental Table S2**:** Contingency table comparing Milioli et al., intrinsic subtype and IHC subtype among 989-case METABRIC cohort.* label named ‘inconsistent’ provided by Milioli et al.^19^

|  | | **Milioli et al., Intrinsic subtype (61.07% agree)** | | | | | | |
| --- | --- | --- | --- | --- | --- | --- | --- | --- |
|  |  | **Basal** | **Her2** | **Inc*** | **LA** | **LB** | **Normal** | **Sum** |
| **IHC**  **subtype** | **TN** | 86 | 40 | 0 | 0 | 0 | 8 | 134 |
|  | **Her2+** | 2 | 55 | 0 | 0 | 0 | 1 | 58 |
|  | **LA** | 0 | 1 | 3 | 158 | 12 | 32 | 206 |
|  | **LB1*** | 6 | 11 | 20 | 226 | 258 | 12 | 533 |
|  | **LB2** | 0 | 3 | 1 | 6 | 47 | 1 | 58 |
|  | **Sum** | 94 | 110 | 24 | 390 | 317 | 54 | 989 |

## Supplemental figures

Supplemental Figure S1**:** Density of *MKI67* gene expression among IHC subtypes in all three datasets in the original scale. (A): In-house RNA-seq. (B): TCGA BC RNA-Seq. (C): METABRIC discovery set.


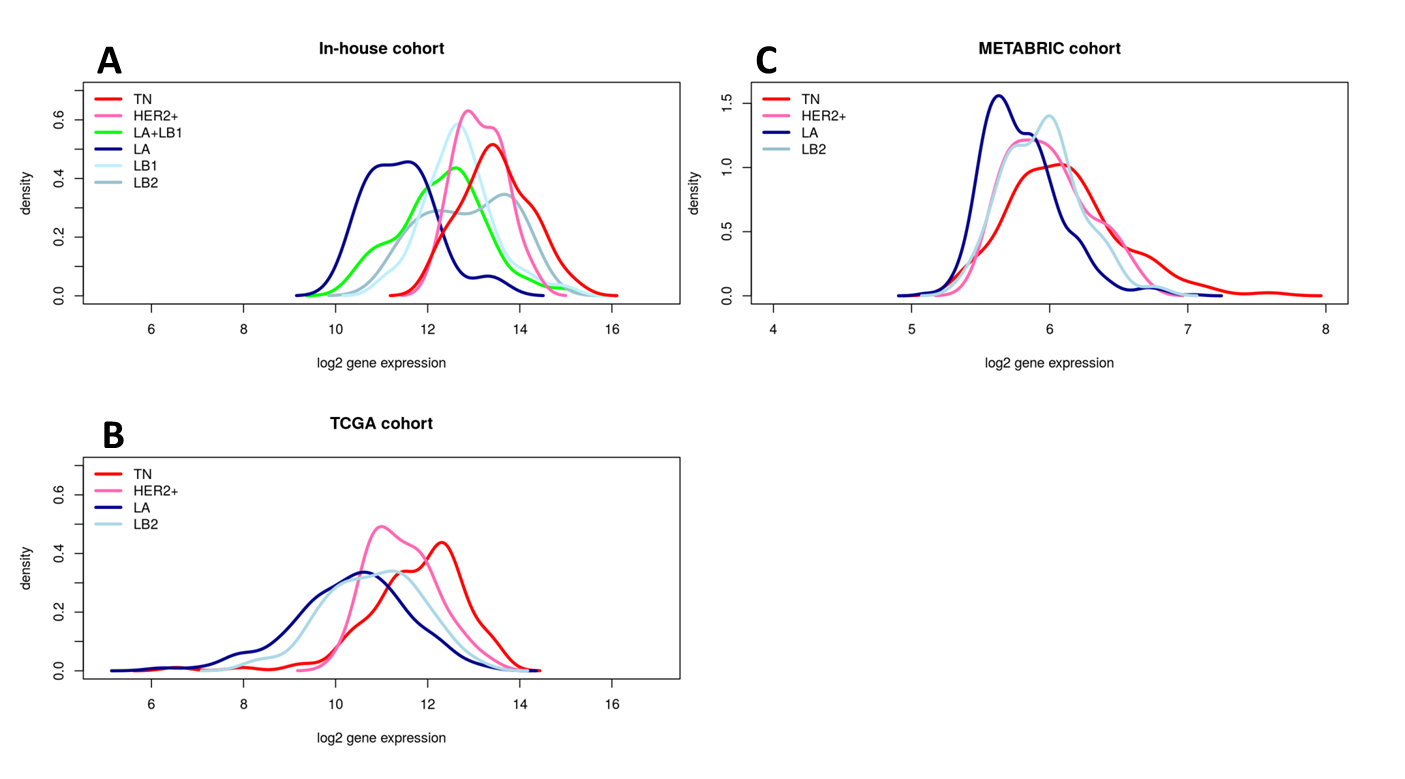


Supplemental Figure S2**:** Percentage of misclassified cases across the PC1 axis for in-house (A), TCGA 712 cohort (B) and METABRIC 989 cohort (C). Vertical line is drawn at the PC1 point with minimum percentage of misclassified cases. In the case of more than one PC1 point with minimum percentage of misclassified cases then the mean of minimum is used. The cutoff for in-house, TCGA and METABRIC cohorts are -0.81, 6.1 and 2.4 respectively.


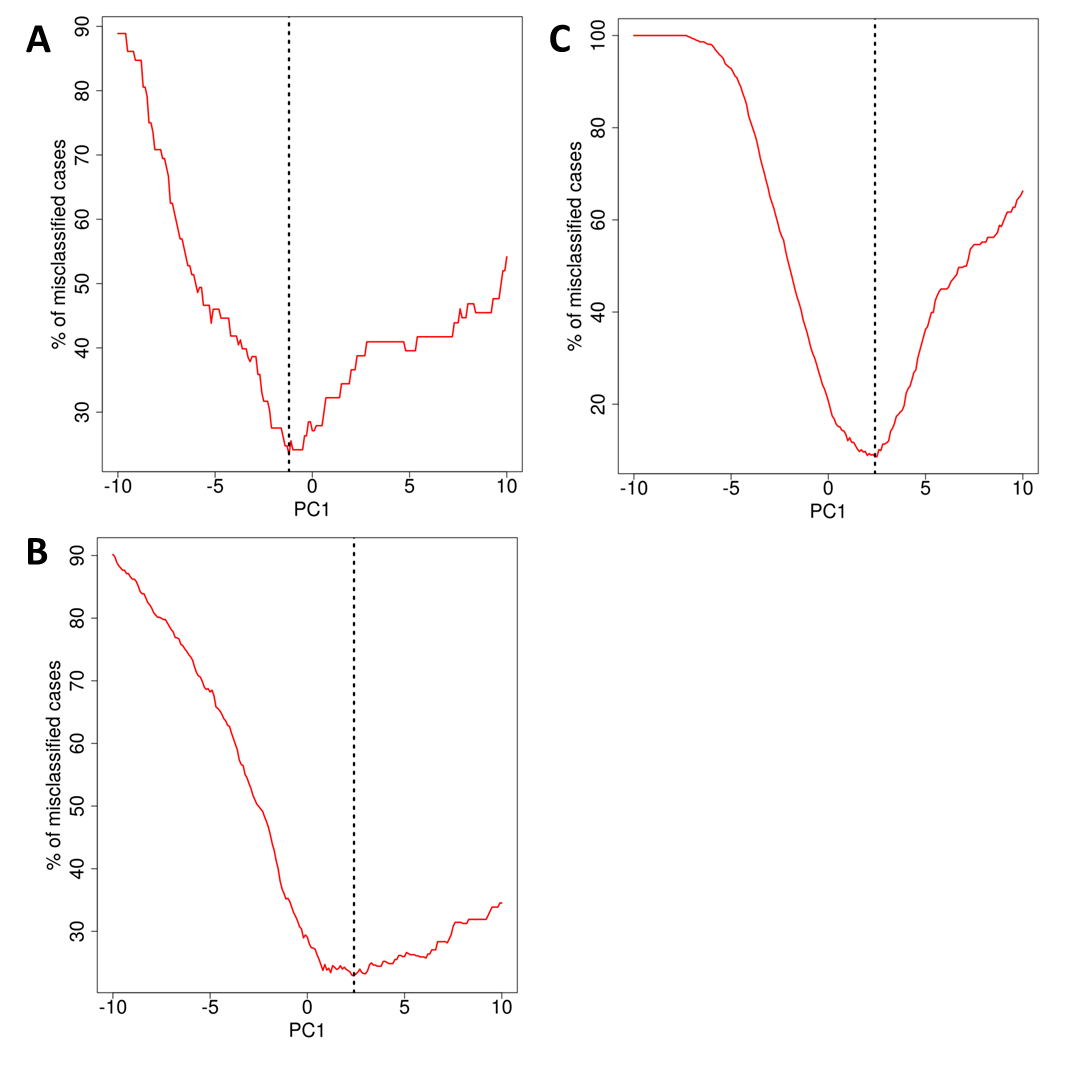


Supplemental Figure S3**:** Comparing *ESR1* gene expression across PC1 axis for in-house (A&B), TCGA 712 (C&D) and METABRIC 989 cohort. A, C and E: Boxplot comparing the *ESR1* gene expression. B, D and F: Density plot comparing *ESR1* gene expression. Wilcoxon test p value is given in the figure.


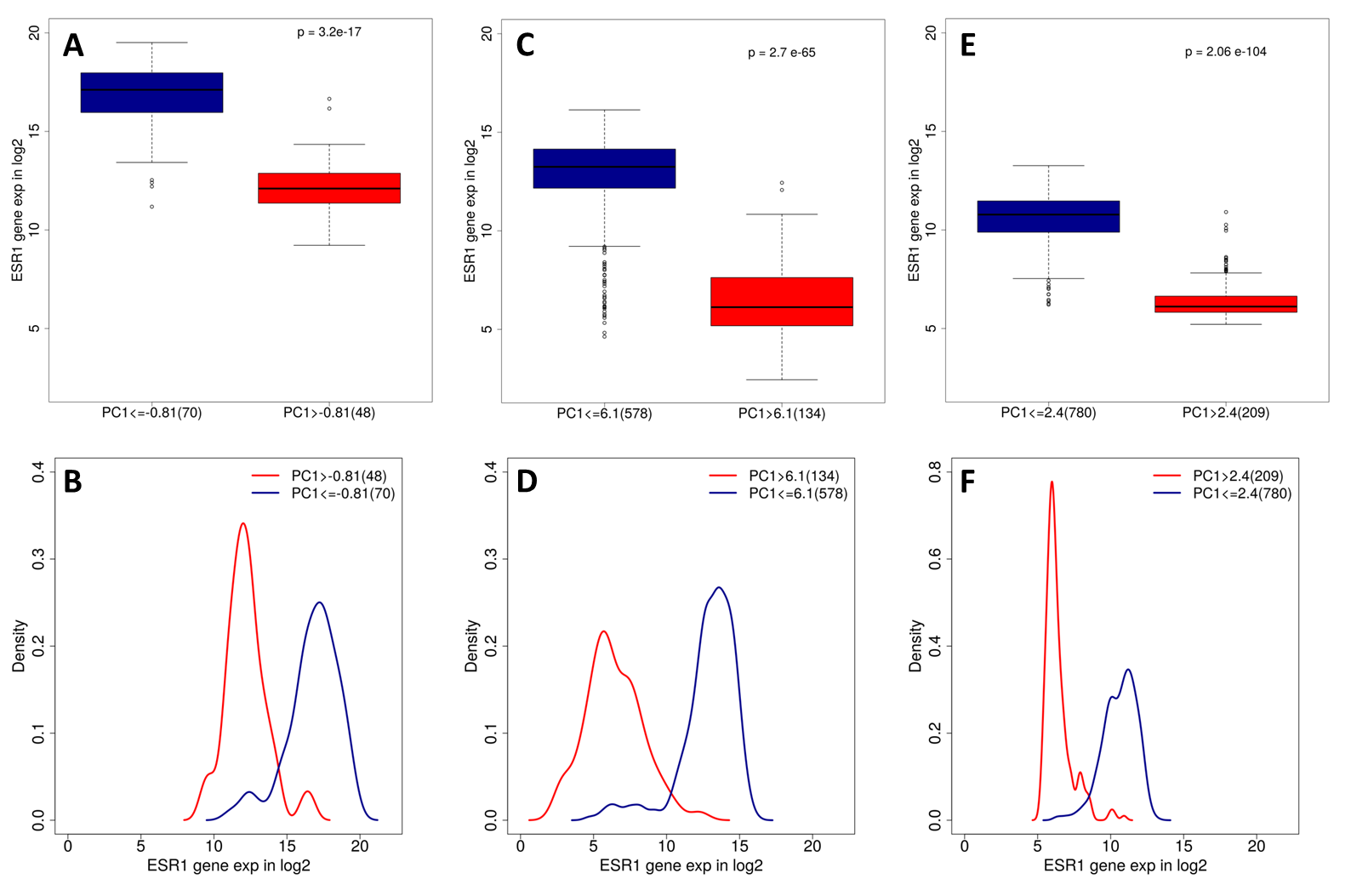


Supplemental Figure S4**:** Comparing *MKI67* gene expression between the cases that switched to LB in refined intrinsic from LA in conventional intrinsic and the cases that are LA in both conventional and refined intrinsic. Boxplot of comparisons for In-house (A), TCGA (B) and METABRIC cohorts (C).


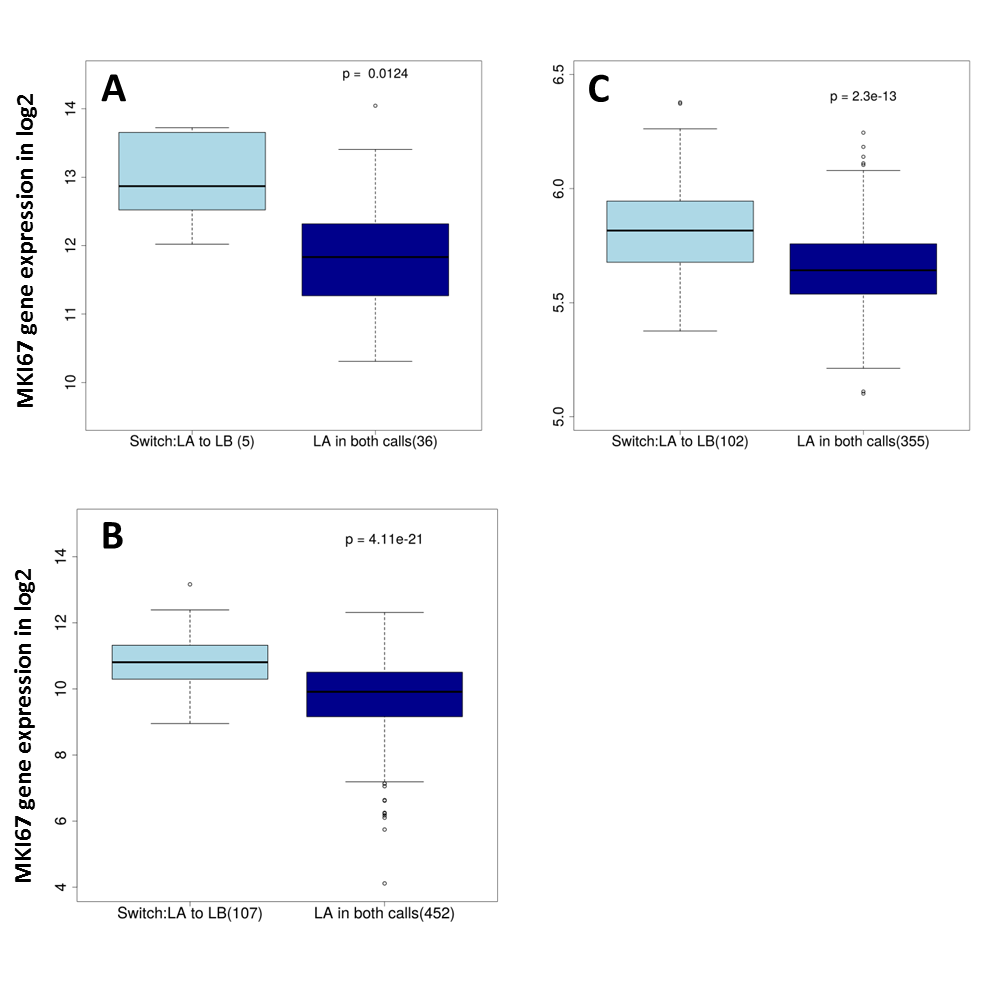


Supplemental Figure S5**:** PCA plot of normalized PAM50 gene expression of METABRIC cohort with refined intrinsic subtype. Vertical line is drawn at the PC1 cutoff.


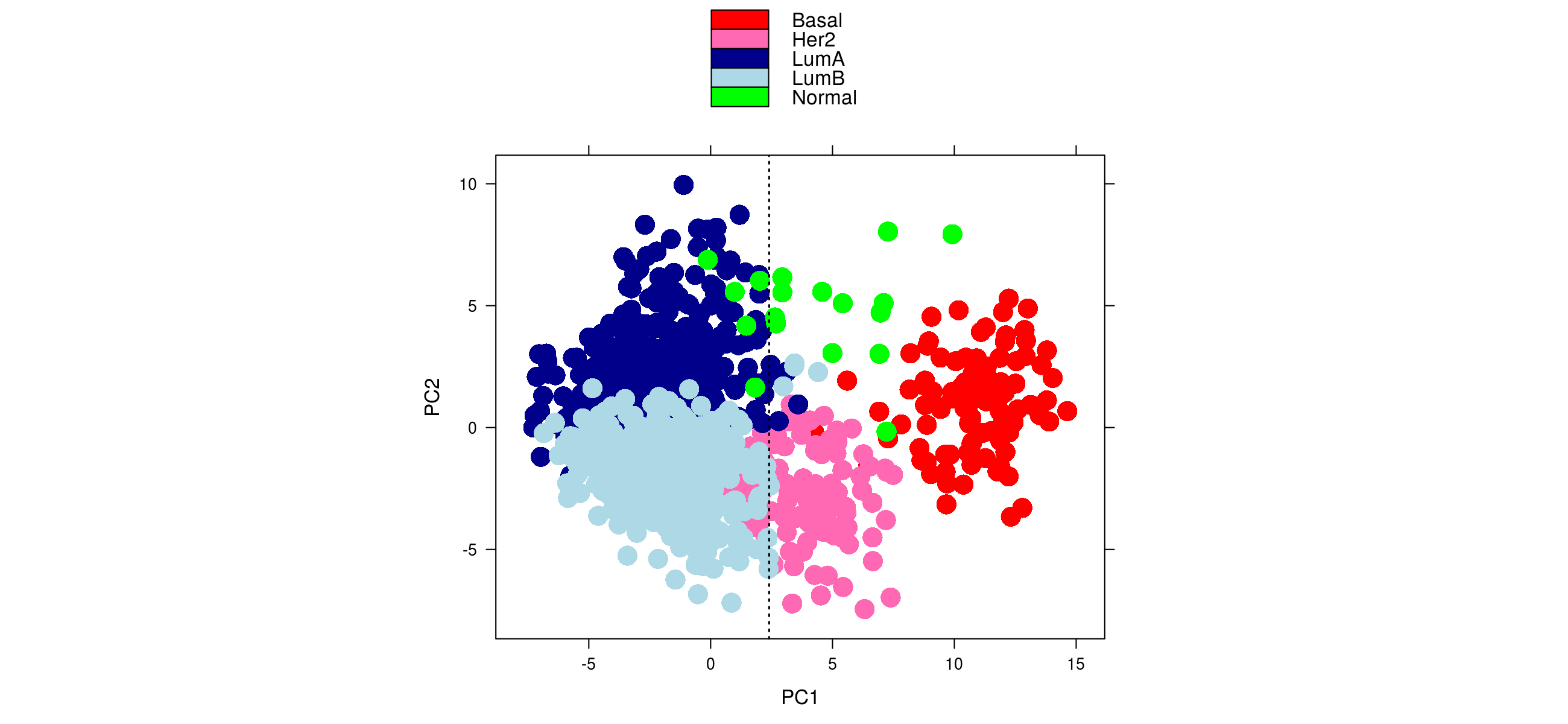


Supplemental Figure S6**:** Difference in survival between the LA and LB subtypes according to IHC subtype in both the TCGA BC (A , C) and METABRIC (B, D) cohort. The Kaplan–Meier curves for cumulative survival in years for LA and LB cohorts defined by IHC for two recommended end points (progression-free interval (PFI) and disease-free Interval (DFI); A, C) in the TCGA BC cohort and for two available end points (disease-specific survival (DSS) and overall survival (OS); B, D) in the METABRIC cohort. P-value, Hazard ratio and the number of events ‘/’ number of cases are given in the legends of plots.


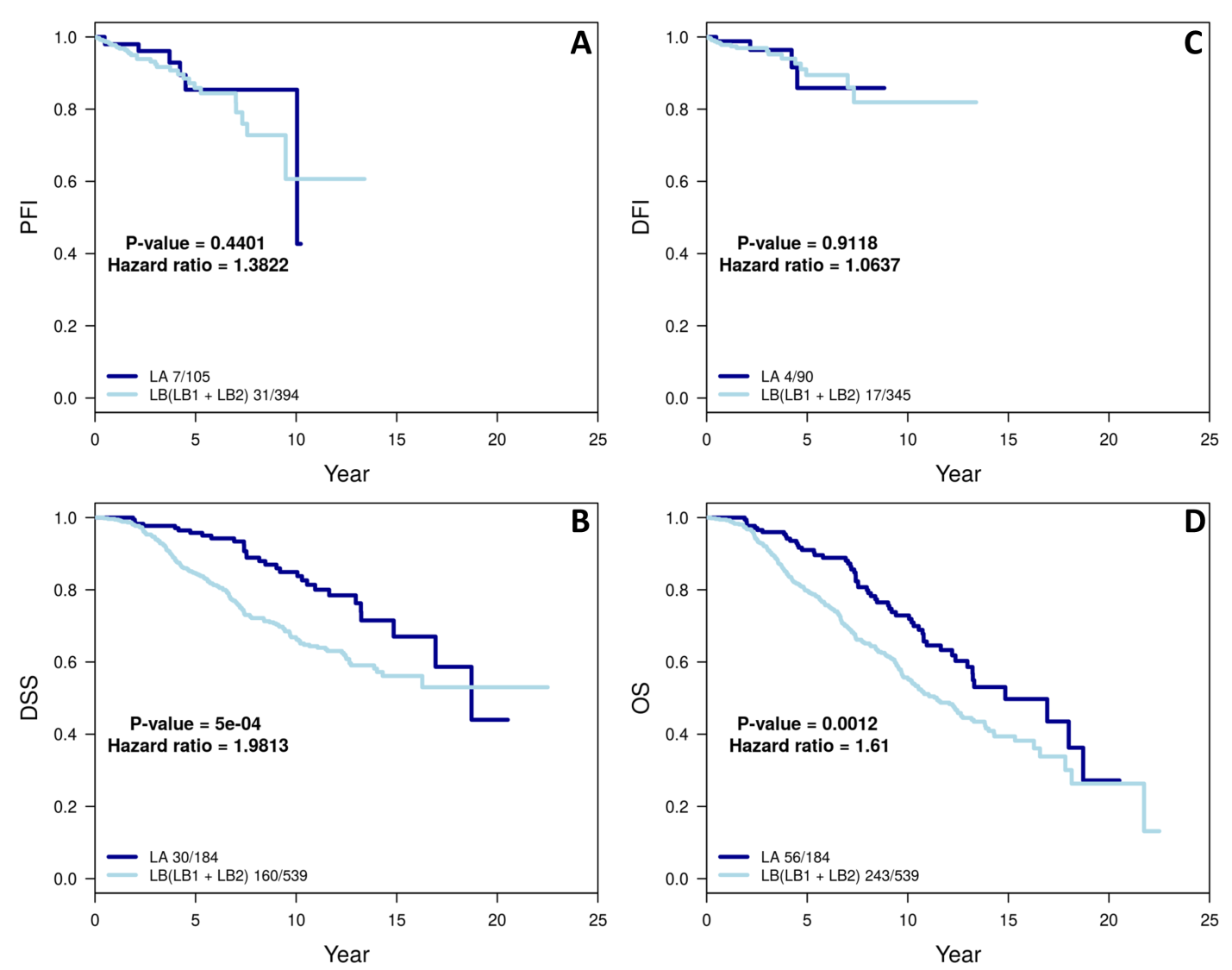

Supplement: Supplementary file 1 — Supplemental Tables and Figures [file 41598_2019_44339_MOESM1_ESM.docx]
